# Inferring Speciation Processes from Patterns of Natural Variation in Microbial Genomes

**DOI:** 10.1093/sysbio/syv050

**Published:** 2015-08-27

**Authors:** David J. Krause, Rachel J. Whitaker

**Affiliations:** Department of Microbiology, University of Illinois at Urbana-Champaign, Urbana, IL 61801, USA

**Keywords:** Barriers to gene flow, differentiation, recombination, selection

## Abstract

Microbial species concepts have long been the focus of contentious debate, fueled by technological limitations to the genetic resolution of species, by the daunting task of investigating phenotypic variation among individual microscopic organisms, and by a lack of understanding of gene flow in reproductively asexual organisms that are prone to promiscuous horizontal gene transfer. Population genomics, the emerging approach of analyzing the complete genomes of a multitude of closely related organisms, is poised to overcome these limitations by providing a window into patterns of genome variation revealing the evolutionary processes through which species diverge. This new approach is more than just an extension of previous multilocus sequencing technologies, in that it provides a comprehensive view of interacting evolutionary processes. Here we argue that the application of population genomic tools in a rigorous population genetic framework will help to identify the processes of microbial speciation and ultimately lead to a general species concept based on the unique biology and ecology of microorganisms.

Although the importance of microbial biodiversity is broadly acknowledged (Falkowski et al. 2008; Turnbaugh et al. 2009), advances in microbial ecology and evolutionary biology are limited without a good understanding of the fundamental units of biology—species (Achtman and Wagner 2008). Molecular tools have shown that microbial diversity appears to be structured into distinct genetic units (Casamayor et al. 2002; Acinas et al. 2004; Sikorski and Nevo 2005; Achtman and Wagner 2008; Koeppel et al. 2008). However, whether these units behave as independent species is unknown. In the absence of good, genome-wide empirical data, contentious debate has been ongoing about what species and speciation mean in microbes, and whether microbial species exist at all (Doolittle and Papke 2006; Papke et al. 2007). Recently, however, the technological innovation of next-generation sequencing coupled with the emerging discipline of population genomics has started to provide a new window on natural variation able to reveal the evolutionary processes ongoing in natural microbial populations. The power of this approach comes from its ability to compare genomic variation across regions of the genome to infer whether different processes are occurring at different loci. Only when these processes are well characterized in a broad diversity of microbial taxa can microbial speciation be understood.

Here we use the unified species concept of de Queiroz: species are “lineages evolving separately from other lineages.” (de Queiroz 2007). With this general definition in mind, we discuss how to detect patterns in microbial genomes to understand the processes that allow microbial lineages to diverge and keep them independent, whether they be clonal species differentiated by selection and/or genetic drift, recombining species isolated by physical barriers, or recombining species that undergo differential ecological selection. We highlight the fact that identifying patterns of genomic variation makes it possible to go beyond the delimitation of species and yields insights into the speciation process, whether it has occurred through ecological selection or because of barriers to gene flow. We therefore see population genomics as an excellent hypothesis-generating tool for examining experimentally the forces driving speciation in natural populations.

The first step in using population genomics to investigate speciation is proper sampling. Because they focus on recent evolutionary events that generate variation among individuals, population genetic studies typically include numerous closely related individuals to avoid the confounding effects of many overlapping events. Because bacteria, archaea, and viruses typically have small genomes, advances in sequencing technology have made it feasible both technologically and financially to sequence the entire genomes of hundreds of individuals. Currently, population genomic methods apply best to cultured individuals isolated from the environment as single laboratory strains so that the linkage between regions of the genome is known. Culture-independent population genomics using bulk environmental DNA has also been attempted (Allen et al. 2007; Eppley et al. 2007; Caro-Quintero and Konstantinidis 2012); however, challenges remain in resolving the linkage of alleles across closely related genomes. These challenges may be overcome by new techniques such as metagenomic chromosome conformation capture (meta3C), a tool which has been shown to aid in the assembly of genomes from bulk environmental DNA (Marbouty et al. 2014). Another new technology on the horizon, single-cell genomics (Kashtan et al. 2014), is likely to enable soon culture-independent analysis of many individual genomes, yet error rates and amplification biases currently prevent this type of study from being widely tractable today.

To identify which individuals should be sequenced, *a priori* hypotheses about the ecological or physical drivers of speciation are often used (Reno et al. 2009; Shapiro et al. 2012). Yet for most microbes the scale at which environmental variation occurs is difficult to determine, making *a priori* hypotheses difficult to generate reliably. As described below, population genomic tools can be used to identify potential sources of differentiation without these *a priori* assumptions, by sequencing as many random individuals within a population as possible. The idea is to randomly partition these sequenced strains into groups and test for patterns of genomic variation indicative of recombination and selection. Because forces such as genetic drift can affect the patterns of variation in natural populations, this sort of *de novo* approach to identifying species within a set of sampled individuals requires great care to distinguish patterns from the null expectation of no differentiation, with corrections for the many comparisons involved in sampling an entire genome. Such an approach may identify novel divisions within a population that do not immediately conform to obvious species boundaries based on known environmental differences (Cadillo-Quiroz et al. 2012). Below we describe the primary models of microbial speciation that have been proposed and the resulting patterns of natural variation predicted to occur in microbial genomes (Fig. 1). A description of commonly used population genetics metrics that may help understand these models in a population genomics framework can be found in Table 1. Once these models are understood, patterns of natural variation can be used to infer the processes of speciation that, although not directly observable, are ongoing in natural populations. These expectations are also summarized in Table 2.

**Figure 1.**
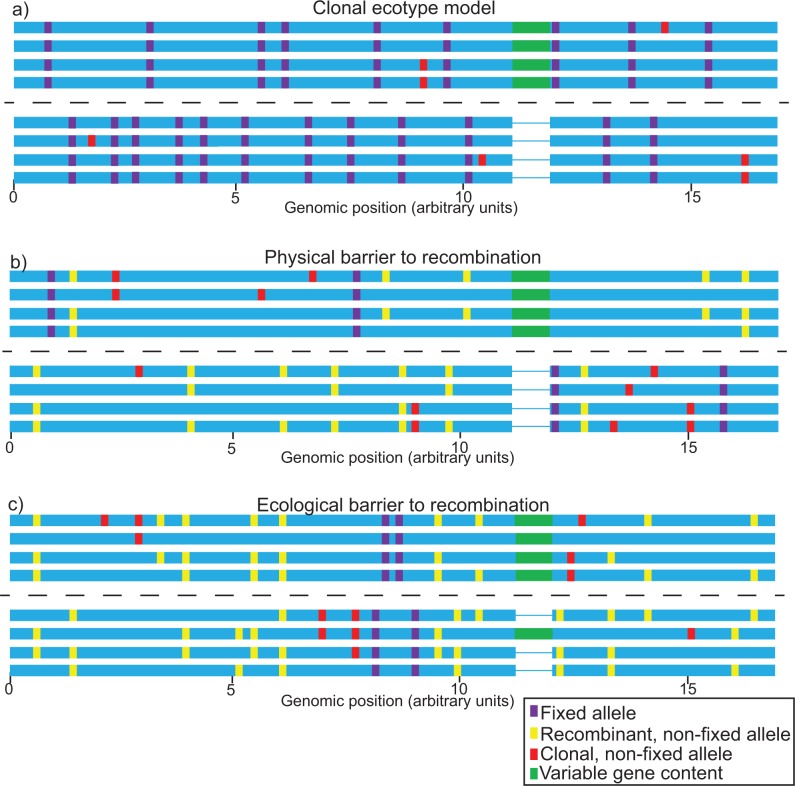
Patterns expected to be detected in population genomic studies of two closely related species for each of three different models of speciation. Horizontal bars represent individual genotypes and colored rectangles indicate polymorphisms within the population: purple rectangles stand for alleles that are fixed in one species and absent in the other one, yellow rectangles represent non-fixed alleles that are recombined between species and are therefore found in both, whereas red rectangles indicate non-fixed alleles that do not recombine but propagate clonally and are therefore only found in one species. Green horizontal rectangles indicate variation in gene content, that is, genes that are present in some individuals (green rectangles) but absent in others (thin lines). Dashed lines separate the two species. a) Patterns derived from the clonal ecotype model, wherein there is no gene flow and SNPs are generally fixed between species. b) Patterns expected in case of a physical barrier to recombination. Recombination occurs within species but not between them, resulting in less fixation than in the clonal ecotype model. c) Patterns expected in case of differential selection with gene flow, in which recombination occurs among all members of the population but strong ecological selection creates islands of divergence between species.

**Table 1.** Description of metrics commonly used in population genomics studies

Metric	Description	Relevance to population genomics
π	Average intraspecific pairwise distance	Nucleotide diversity within a species
${\text{D}}_{\text{XY}}$	Average interspecific pairwise distance	Divergence between species
${\text{F}}_{\text{ST}}$	1–π/${\text{D}}_{\text{XY}}$	Fixation index
$d_{N}$/$d_{S}$	Ratio of non-synonymous to synonymous substitution rates	Detection of positive or purifying selection
Tajima's D	Comparison of intraspecific nucleotide diversity and number of segregating sites	Detection of outlier loci affected by selection

**Table 2.** Three of the main models of speciation for microbes, their supporting evidence that can be found in genomes, and how species can be discerned

Model of speciation	Resulting genomic pattern	How to delineate species
Clonal ecotype model	Little to no evidence for recombination; high ${\text{F}}_{\text{ST}}$ across all genomic loci	Fixed gene content or fixed polymorphism
Physical barriers to recombination	Recombination among some but not all individuals	Recombination rate higher within than between species
Ecological barriers to recombination	Recombination detectable among all individuals in the population; isolated peaks in the ${\text{D}}_{\text{XY}}$ and ${\text{F}}_{\text{ST}}$ profiles	Grouping based on ${\text{F}}_{\text{ST}}$ and ${\text{D}}_{\text{XY}}$ at the peaks of genomic diversity

## What Are the Patterns Predicted to Occur in the Genomes of Microbial Species?

**Figure 2.**
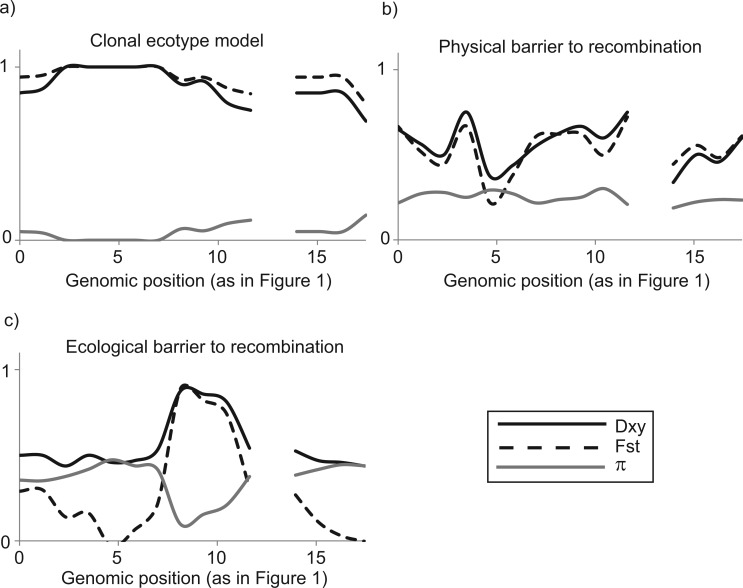
Profiles of intraspecific nucleotide diversity (π), interspecific divergence (${\text{D}}_{\text{XY}})$, and fixation index (${\text{F}}_{\text{ST}})$ predicted to occur in three models of speciation. The three cases correspond to those in Fig. 1: a) clonal ecotype model, b) physical barriers to gene flow, c) differential selection with gene flow. The solid gray lines represent π, the solid black lines ${\text{D}}_{\text{XY}}$, and the dashed lines ${\text{F}}_{\text{ST}}$ between species. Gaps in the plot correspond to the position of variable gene content. Values are based on the theoretical patterns of Fig. 1.

One of the most widely accepted models for microbial species is the clonal ecotype model, in which an “ecotype” is defined as “a group of bacteria that are ecologically similar to one another, so similar that genetic diversity within the ecotype is limited by a cohesive force, either periodic selection or genetic drift, or both” (Cohan and Perry 2007). This model posits that, because microorganisms are clonally reproducing, recombination rarely occurs among individuals within a population (Cohan 2002). In such a situation, periodic selective sweeps of adaptive, niche-specific mutations purge genomic diversity within the ecotypes. Independent selective sweeps occurring in different ecotypes result in a low genomic diversity within species but a high fixed divergence between them that increases over the time that they are isolated from one another. This type of pattern can be identified in genomic data using ${\text{F}}_{\text{ST}}$, the fixation index, which measures population differentiation due to genetic structure (Hartl 2007). It can be calculated using the formula ${\text{F}}_{\text{ST}}=1-\pi /{\text{D}}_{\text{XY}}$ (Hudson et al. 1992), where π is the intraspecific nucleotide diversity (the average number of nucleotide differences between two individuals sampled randomly within a species; Nei and Li 1979) and ${\text{D}}_{\text{XY}}$ is the interspecific divergence (the average number of nucleotide differences between species). The clonal ecotype model predicts that fixed differences (${\text{F}}_{\text{ST}}$ values close to 1) should occur throughout the genome (Fig. 2): if selective sweeps occur frequently within a population, they decrease π and increase ${\text{D}}_{\text{XY}}$, resulting in ${\text{F}}_{\text{ST}}$ values close to 1 (Nei 1987). Some versions of the clonal ecotype model allow for horizontal gene transfer of novel gene content into clonal backgrounds; in a primarily clonal context, these genes may become fixed within a population if they are adaptive or in close linkage with a positively selected mutation (Tettelin et al. 2005; Koeppel et al. 2008).

In the clonal ecotype model, genome-wide divergence and fixation are predicted to occur throughout the genome and not solely at the loci responsible for speciation. However, it may be possible to identify these loci under some conditions. For instance, the periodic selection events that underlie the clonal ecotype model may at the same time promote key amino acid changes responsible for species divergence and purge neutral diversity, leading to elevated ratios of non-synonymous ($d_{N}$) to synonymous ($d_{S})$ substitution rates specifically at the loci experiencing selection (Allen et al. 2007).

Outside of the clonal ecotype model there also exist models of speciation in microbes that allow a larger role for recombination. Based on patterns of natural variation in multi-locus sequence typing (MLST) studies, it has been suggested that recombination in microorganisms may overcome clonal reproduction under some conditions (Hanage et al. 2005; Whitaker et al. 2005), and both homologous and non-homologous recombination can be detected across the genome by looking at patterns of genome sequence (Didelot et al. 2010; Krause et al. 2014). In this case the clonal ecotype model and its derivatives are not suited: instead, new models that incorporate recombination must be used. Like in sexual eukaryotes, speciation in recombining microorganisms requires barriers to gene flow. Well-studied mechanisms that can cause this include decreases in homologous recombination when sequence divergence increases (Greig et al. 2003; Fraser et al. 2007) as well as physical separation caused by geographic isolation (Whitaker 2006; Whitaker et al. 2003). In what follows, we base ourselves on the literature on sexual eukaryotes to describe the patterns expected to be found in the genomes of recombining microorganisms experiencing speciation due to physical barriers or adaptive divergence (Nosil and Feder 2012).

The possibility that geographic barriers to gene flow may cause allopatric speciation in some microbial systems was previously studied at the MLST level (Whitaker et al. 2003; Vos and Velicer 2008). Such physical barriers to gene flow may be identified if recombination is frequent within particular groups of strains but limited between them. Species formed by physical barriers to gene flow present genomic signatures of high ${\text{F}}_{\text{ST}}$ and ${\text{D}}_{\text{XY}}$ between species; however, unlike in the clonal model, π is relatively high and constant across the genome of each species since periodic selection is counteracted by recombination (Begun and Aquadro 1992; Fig. 2). In the absence of physical barriers, selection in different environments may differentiate lineages and promote ecological speciation. In cases where recombination rate is high and constant across the genome, theory predicts that most genome regions will display low ${\text{F}}_{\text{ST}}$ and low ${\text{D}}_{\text{XY}}$ However, speciation loci will stand out as “islands” characterized by high differentiation (${\text{F}}_{\text{ST}}$), high interspecific divergence (${\text{D}}_{\text{XY}}$), and low intraspecific nucleotide diversity (π) (Fig. 2; Nadeau et al. 2012; Nosil and Feder 2012; Via 2012). In theory, if ecological differences between nascent species result in preferences for different habitats, species will diverge and become ecologically differentiated. Evidence for ecological selection may be observable in speciation islands, just like in the clonal ecotype model, using metrics such as $d_{N}/d_{S}$ In addition, other metrics such as Tajima's D, which compares intraspecific nucleotide diversity to the number of segregating sites within a species (Tajima 1989), can be used. This metric is sensitive to other population effects such as bottlenecks, which affect the entire genome, and is therefore most useful to detect outlier loci in recombining genomes.

Ultimately, the different models proposed for microbial speciation are likely to result in very different genomic patterns. However, as we will see, testing these various models and determining which one is best suited to a particular system requires a thorough approach that includes measurements of relative and absolute divergence, tests for selection, and inferences of gene flow.

## What are the Patterns Detected in Microbial Genomes?

Very few studies have put all the pieces together by testing for gene flow, selection, diversity and divergence in a large set of genomes. The best data in support of the clonal ecotype model come from experimental evolution studies, which are performed under strict laboratory conditions where recombination is limited or precluded. In these studies, the genomic signatures of the periodic selection events that are the cornerstone of the ecotype model can be analyzed immediately following their occurrence. The best example is Lenski's long-term evolution experiment, in which *Escherichia* *coli* cultured in laboratory medium for more than 50,000 generations have been analyzed phenotypically as well as subjected to genomic analyses (Wiser et al. 2013). An analysis of the increasing fitness of clones over tens of thousands of generations in the lab, suggesting that many selective sweeps occurred, found 26 fixed mutations in coding regions of evolved strains after 20,000 generations, all of which were non-synonymous substitutions (Barrick et al. 2009). A compelling example of ecotype divergence occurred naturally within this long-term experiment when a divergent lineage emerged with a novel ability to metabolize citrate, a previously unexploited carbon source present in the medium (Blount et al. 2008). In this scenario, the ecological differentiation was tied to the tandem amplification of a gene involved in citrate transport, as well as to a few potentiating and actualizing mutations specific to this lineage (Blount et al. 2012). In these flasks, two lineages, one utilizing glucose and the other utilizing citrate, are stably coexisting and likely to persist through future generations.

In natural populations, many studies have assumed that bacteria are primarily clonal and have used comparative genomics to find evidence of ecological differentiation based on ecological niches defined *a priori* For example, a broad study of *Prochlorococcus marinus* sequenced genomes of strains from ecotypes adapted to high or low levels of light (Paul et al. 2010). When looking at $d_{N}/d_{S}$ ratios between strains adapted to different light levels, the authors detected 78–90 genes that appeared to be under positive selection. However, 68 genes were found to be under positive selection when the comparison was made between low-light ecotypes, implying some alternative ecological differentiation that was not identified *a priori*. Although this study was able to identify the genetic basis of ecological differentiation, it did not pinpoint the cause of the speciation process because the ecotypes investigated were highly divergent by population standards and some variables such as gene flow and genetic drift were not investigated.

**Figure 3.**
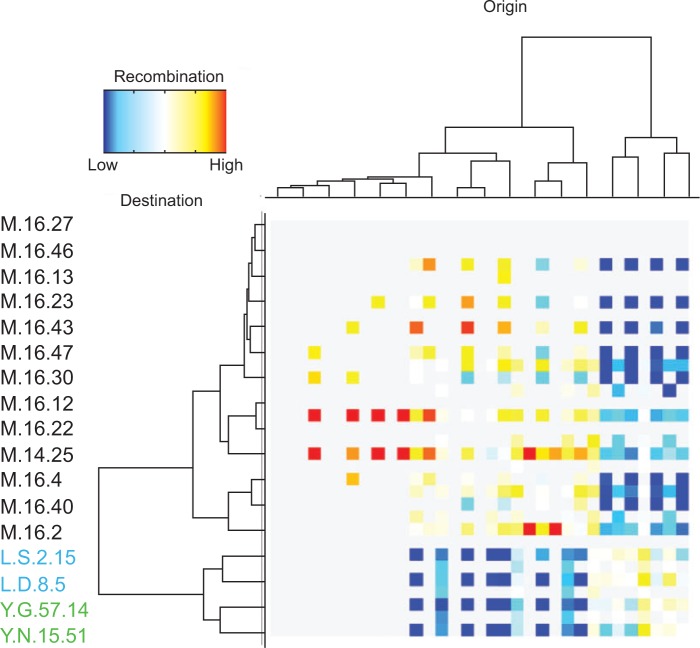
Analysis of the influence of geographical barriers on patterns of gene flow in *Sulfolobus* using ClonalOrigin (Didelot et al. 2010). This program starts by reconstructing a single clonal phylogeny of the strains (shown on the left and on top of the diagram), then determines the frequency of recombination events between each pair of strain (including the inferred ancestor strains), which are depicted in the diagram as lower than expected (blue), not significantly different from expectation (white) or higher than expected (red). Strain names in black and starting with the letter “M” were isolated from Kamchatka (Russia). Strain names in blue (L.S.2.15, L.D.8.5) come from Lassen National Park (California), and those in green (Y.G.57.14, Y.N.15.51) come from Yellowstone National Park (Wyoming).

In species that are known to recombine more frequently, patterns of differentiation among genomes have yielded information on physical and ecological barriers. Physical barriers that isolate populations were identified in divergent populations of the archaeaon *Sulfolobus islandicus* collected from hot springs in Russia, North America, and Iceland (Whitaker et al. 2003), and limited gene flow among these populations has been found across both the core (Fig. 3) and the variable parts of their genomes (Reno et al. 2009). Other physical barriers to gene flow may occur in sympatry; for example, strains of *S. islandicus*, isolated from a single hot spring, were found to belong to two species with rates of recombination higher within than between them (Cadillo-Quiroz et al. 2012). In this case there was no clear target for differential selection. The authors of this study hypothesized that physical barriers to gene flow (resulting from incompatible transfer mechanisms such as pili and surface layer components) or *in situ* ecological differences (resulting from adaptation to microenvironments) may be responsible for this apparent case of sympatric speciation.

In the microbial eukaryote *Neurospora crassa*, recently diverged but recombining populations living either off the Louisiana coast or in the Caribbean were analyzed for evidence of ecological differentiation among incipient allopatric species. A population genomic approach using ${\text{F}}_{\text{ST}}$ to measure fixation between species, ${\text{D}}_{\text{XY}}$ to measure divergence, and Tajima's D to identify selection found two genomic regions that could explain population differentiation (Ellison et al. 2011). Further searches for signatures of selection identified allelic differences in one of these regions, containing a RNA helicase gene that may be involved in the differentiation.

In the bacterium *Vibrio cyclitrophicus*, species were identified in which ecological differences (namely, association with particles of different sizes) rather than physical barriers appear to be driving speciation (Hunt et al. 2008). Analysis of genome sequences found evidence for fixation between two species in four small genomic islands against a background of high levels of gene flow in the rest of the genome, suggesting that these four regions are involved in the differentiation between these species (Shapiro et al. 2012). Also, there was evidence for more frequent recombination within species than between them, suggesting that the observed ecological differentiation may also have resulted in a barrier to gene flow. In a study of *Bacillus cereus* *sensu lato*, several clades of which exhibit different phenotypes with respect to pathogenicity, recombination was inferred from genome sequences to be 1.9–4.3 times more frequent than mutation, but a deep ancestral divergence between clades was still present (Zwick et al. 2012). Analysis of clade-specific selection using $d_{N}/d_{S}$ ratios identified candidates for differentiation in some highly conserved genes, specifically genes involved in amino acid metabolism. Although the relative rates of intraspecific and interspecific recombination were not investigated, these results suggest that ecological speciation is occurring at these loci.

Because barriers caused by physical factors such as geographic separation create effects similar to those of barriers caused by ecological differentiation, it may be difficult to distinguish between the two. Also, inferences may differ depending on whether we are looking at an incipient speciation or at a well-established one (Shapiro and Polz 2014). Islands of speciation may only be present during early divergence, as in the *Vibrio* study (Shapiro et al. 2012; Shapiro and Polz 2014). Much larger “continents” of fixation, as in the *Sulfolobus* study (Cadillo-Quiroz et al. 2012), may be observed later in the process of speciation or might form neutrally if the barriers to recombination between species are not acting across the whole genome. Regions experiencing low recombination are the first to diverge while regions that recombine frequently maintain shared polymorphisms and experience little divergence and fixation (Nachman and Payseur 2012). In this case, the regions of low recombination become fixed not because of ecologically driven divergence between species or because of selective sweeps but because of background selection, as observed in *S. islandicus* species (Krause et al. 2014).

## Where Do We Go Next?

Because evolutionary forces interact in nature, teasing apart the inferred processes responsible for the patterns observed in population genomics is a difficult task. Measurements of parameters such as selection coefficients, relative fitness, and recombination and mutation rates in the lab are often needed to clarify the interpretation of the patterns observed in natural populations. In addition, although it is impossible to replay evolution in a natural setting, the patterns predicted to result from various speciation processes can be tested in the laboratory using microbial populations. Even though cultivable organisms make up a small fraction of microbial diversity, most strains currently studied in population genomics are derived from wild strains isolated in the lab. This opens the door to laboratory-based investigations that will supplement our knowledge of species in nature.

The physiological basis of ecological differentiation may also be studied in laboratory populations. Lab-derived ecotypes of *E. coli* that utilize different resources show highly divergent gene expression profiles, which implies that divergence may not only be found in genes responsible for differentiation but also in their regulators (Kinnersley et al. 2009). A recent study using laboratory microcosms of *Bacillus subtilis* clones found that, in a majority of replicate cultures, distinct putative ecotypes evolved that differed by colony morphology and competitive growth rates (Koeppel et al. 2013). These studies illustrate the potential for ecotypes to form even under controlled laboratory growth conditions. Comprehensive genomic analyses of the resulting ecotypes would do much to illuminate genome-level processes of ecotype formation, especially since these analyses can be performed immediately following differentiation.

For recombining organisms we are likely to be able to find out the genetic sources of ecological differentiation. The true gold standard of delineating ecological species in these organisms will come from using genomics to identify genetic loci under selection then performing controlled tests to explain the function of these genes in their ecological context. This is likely to be easier for some organisms than for others. In the case of well-studied model organisms such as *E. coli*, the function of many genes is well understood, making it far easier to come by hypotheses; besides, these strains are generally amenable to genetic manipulation, making them prime targets for laboratory study (Luo et al. 2011). In the case of *Neurospora*, for instance, some understanding of the role of RNA helicases in adaptation to cold was needed to formulate a hypothesis for how one RNA helicase gene might be responsible for the differences observed between species (Ellison et al. 2011). These types of conclusions will be harder to reach for organisms less well understood, such as the uncultured microbial majority; besides, when validating speciation targets obtained from genome scans one should not content oneself with a narrative based on functional annotation (Pavlidis et al. 2012). The strength in utilizing such genome-wide association studies lies in their ability to identify genes that are under selection without any prior gene characterization. Investigators need not, nor are they able to, make assumptions about what types of genes are likely to become differentiated in certain environments.

When searching for loci responsible for ecological speciation, speciation islands are impossible to identify if species delimitations are based on average nucleotide identity or other sequence-based clustering analysis, because such approaches, by definition, only detect patterns of divergence if they are shared by a majority of loci. Identifying recombination patterns and delimiting potential biological species *a posteriori* based on these patterns may be the best way to identify truly significant ecological boundaries. Without some barrier to recombination, organisms cannot diverge into separate species. In addition, statistical analyses are needed to detect outlier loci more differentiated than the rest of the genome, especially when conflicting evidence supports alternative species assignments. Although genomic evidence can be used to confirm *a priori* species delimitations, letting genomic data speak for themselves is a better approach as it can identify species boundaries that were not expected. Indeed, microbial ecotypes may not be delineated by parameters believed to be important such as temperature, pH, or salinity; biology always has the capacity to surprise!

When biological species are found, further experimental examination is required to find out the mechanism responsible for speciation. For example, a better understanding of the mechanisms of recombination is required to predict where physical barriers to gene flow might occur. Straightforward tests for detecting barriers to recombination should be performed to find out whether speciation is caused by physical barriers rather than by ecological ones. Patterns such as those described for *S. islandicus* strains (Fig. 3) may result from a variety of mechanisms of physical isolation. In sympatry, physical barriers to recombination can result from a diversity of mechanisms including restriction modification, CRISPR-Cas systems, and decreased homologous recombination between divergent sequences (Fraser et al. 2007; Doroghazi and Buckley 2011). Some naturally competent bacteria also have specific signal sequences responsible for the uptake of DNA closely related to the host genome, thereby increasing the level of gene flow among closely related strains (Redfield et al. 2006). Finally, antagonistic interactions, common among microorganisms, can also define routes for gene exchange among species (Cordero et al. 2012). All of these mechanisms can be investigated to some extent in the laboratory.

Experimental methods to detect barriers to gene flow include sorting cells into microcosms and periodically looking for evidence of recombination, or establishing genetically tractable cultures in the laboratory and using genetic crosses to investigate recombination barriers (Zhang et al. 2013). Also, the potential for varying recombination rates to alter the speed at which different parts of the genome undergo differentiation requires further investigation. Variations in recombination rates in different chromosomal regions have been identified in some microorganisms (Touchon et al. 2009; Krause et al. 2014). Besides, genomic analyses of microorganisms often reveal that intragenomic recombination is enhanced at loci involved in processes such as immune evasion, cellular defense, cell wall formation and motility (Haven et al. 2011; Caro-Quintero and Konstantinidis 2012). Direct measurement of genome-wide recombination rates in the laboratory has long been a hallmark of eukaryotic biology, but it is currently being initiated in bacteria such as *Streptococcus, Haemophilus, Pneumococcus*, and *Mycobacterium* (Brochet et al. 2008; Mell et al. 2011; Croucher et al. 2012; Gray et al. 2013; Mell et al. 2014). Laboratory experiments offer unique controlled environments to isolate and test specific hypotheses.

Population genomics is turning into an indispensable tool for microbiologists because it allows studying variation beyond what even the most powerful microscopes can see. However, as new data continue to pour in, it is becoming clearer and clearer that even population genomics is unlikely to solve the problem of microbial species all by itself. Given the immense diversity of the microbial world, microbiologists should not expect that whole-genome sequences will help them converge upon some critical threshold of nucleotide similarity distinguishing interspecific diversity from intraspecific variation; rather, population genomics will reveal the processes through which species form. We need to continue to apply our understanding of bacteria, archaea, and eukaryotes to conceive and explain how species are formed and maintained, embracing the diversity among all three domains. Where possible, observations from laboratory experiments or mesocosm studies are required to confirm the hypotheses derived from genomic studies. For cultivable organisms, genes involved in ecological differentiation can be used in genetic tests to demonstrate association with the expected phenotypes of distinct ecotypes. Barriers to recombination can be discovered and further investigated using genetic crosses in the laboratory. Current research using genomic approaches to investigate microbial species has the exciting allure of setting strong precedents for future work, but with this comes a strong responsibility to properly utilize the tool sets and explore all theoretical options. Despite the many drawbacks of studying organisms that cannot be easily seen, the synthesis of population genomics with evolution and molecular biology is likely to bring a better understanding of species in the microbial world.
